# Association between Selected Oral Pathogens and Gastric Precancerous Lesions

**DOI:** 10.1371/journal.pone.0051604

**Published:** 2013-01-07

**Authors:** Christian R. Salazar, Jinghua Sun, Yihong Li, Fritz Francois, Patricia Corby, Guillermo Perez-Perez, Ananda Dasanayake, Zhiheng Pei, Yu Chen

**Affiliations:** 1 Department of Epidemiology and Health Promotion, New York University College of Dentistry, New York, New York, United States of America; 2 Department of Epidemiology, Columbia University Mailman School of Public Health, New York, New York, United States of America; 3 Department of Basic Science and Craniofacial Biology, New York University College of Dentistry, New York, New York, United States of America; 4 Department of Medicine, New York University School of Medicine, New York, New York, New York, United States of America; 5 New York University Cancer Institute, New York University School of Medicine, New York, New York, New York, United States of America; 6 Department of Periodontology and Implant Dentistry, New York University College of Dentistry, New York, New York, New York, United States of America; 7 Division of Epidemiology, Department of Environmental Medicine, New York University School of Medicine, New York, New York, New York, United States of America; 8 Department of Microbiology, New York University School of Medicine, New York, NY, United States of America; 9 Department of Pathology, New York University School of Medicine, New York, New York, New York, United States of America; University of Southern California, United States of America

## Abstract

We examined whether colonization of selected oral pathogens is associated with gastric precancerous lesions in a cross-sectional study. A total of 119 participants were included, of which 37 were cases of chronic atrophic gastritis, intestinal metaplasia, or dysplasia. An oral examination was performed to measure periodontal indices. Plaque and saliva samples were tested with real-time quantitative PCR for DNA levels of pathogens related to periodontal disease (*Porphyromonas gingivalis, Tannerella forsythensis, Treponema denticola*, *Actinobacillus actinomycetemcomitans*) and dental caries (*Streptococcus mutans* and *S. sobrinus*). There were no consistent associations between DNA levels of selected bacterial species and gastric precancerous lesions, although an elevated but non-significant odds ratio (OR) for gastric precancerous lesions was observed in relation to increasing colonization of *A. actinomycetemcomitans* (OR = 1.36 for one standard deviation increase, 95% Confidence Interval = 0.87–2.12), *P. gingivalis* (OR = 1.12, 0.67–1.88) and *T. denticola* (OR = 1.34, 0.83–2.12) measured in plaque. To assess the influence of specific long-term infection, stratified analyses by levels of periodontal indices were conducted. *A. actinomycetemcomitans* was significantly associated with gastric precancerous lesions (OR = 2.51, 1.13–5.56) among those with ≥ median of percent tooth sites with PD≥3 mm, compared with no association among those below the median (OR = 0.86, 0.43–1.72). A significantly stronger relationship was observed between the cumulative bacterial burden score of periodontal disease-related pathogens and gastric precancerous lesions among those with higher versus lower levels of periodontal disease indices (p-values for interactions: 0.03–0.06). Among individuals with periodontal disease, high levels of colonization of periodontal pathogens are associated with an increased risk of gastric precancerous lesions.

## Background

Gastric cancer is the fourth most common cancer and second highest in mortality worldwide [1]. In 2008, there were approximately 989,000 new gastric cancers diagnosed globally and 738,000 deaths. While environmental and lifestyle factors including *H. pylori* colonization, cigarette smoking, dietary intake of salt and preserved foods, and low intake of Vitamin C and vegetables, all contribute to the etiology of the malignancy [2]–[4], these risk factors could only explain less than 60% of gastric cancer incidence [5].

For the most common “intestinal” subtype of gastric adenocarcinomas, which account for approximately 70% of gastric cancer cases [2], [6], a pre-neoplastic sequence has been defined from chronic superficial gastritis through atrophic gastritis, intestinal metaplasia, and ultimately to dysplasia and adenocarcinoma [7], [8]. Studies of gastric precancerous lesions can be used to identify risk factors that play a role in the development of gastric cancer. Several case-control studies of gastric precancerous lesions, predominantly atrophic gastritis and intestinal metaplasia, have identified common risk factors for gastric cancer such as cigarette smoking, old age, and low levels of fruit or vitamin C intake [9]–[14].

The association of self-reported tooth loss or poor oral hygiene with increased risk of gastric cancer has been reported in several epidemiologic studies with diverse populations [15]–[19]. We have previously reported a significant positive association between active gingival bleeding and presence of gastric precancerous lesions in a cross-sectional study of an endoscopic population [20], consistent with the hypothesis that periodontal disease may increase the risk of gastric cancer. However, these studies lack information on specific oral pathogens necessary to elucidate the role of oral health in gastric cancer development. The investigation of specific oral pathogens related to gastric cancer or gastric precancerous lesions may help identify the underlying mechanisms and generate knowledge that may lead to improved clinical treatment and interventions. In this study, we examined the association between selected oral pathogens and presence of gastric precancerous lesions. We considered microbes that are etiologically linked with periodontal diseases [21], including *Porphyromonas gingivalis*, a gram-negative oral anaerobe commonly associated with adult periodontal disease [22]; *Tannerella forsythensis*, an anaerobic gram-negative member of the Cytophaga-Bacteroides family implicated in periodontal diseases, *Treponema denticola*, a motile and highly proteolytic bacterium associated with the incidence and severity of human periodontal disease, and *Actinobacillus actinomycetemcomitans*, a gram-negative facultative anaerobe, which is not only a typical cause of periodontitis but also associated with systemic infections [23], [24]. We also measured levels of *Streptococcus mutans* and *S. sobrinus*, gram-positive bacteria, which are significantly associated with tooth decay [25], [26].

## Methods

### Study population

A detailed description of the study has been presented elsewhere [20]. Briefly, subjects ≥40 years of age undergoing an upper endoscopy at Bellevue Hospital Center in New York City were invited to participate in the study. Information about demographic and lifestyle factors was collected using structured questionnaires administered by a trained interviewer.

Serum samples were collected at the time of endoscopy and were tested for *H. pylori* using ELISA for IgG antibodies to *H. pylori* whole cell antigens in duplicate and in parallel with known positive controls (cutoff for positivity was an optical density ratio >1.0 [27]). IgG antibodies to *cagA* were measured by ELISA (the cutoff for positivity was an optical density ratio >0.35 [28]).

Endoscopic evaluation was performed using Olympus GIF-XQ140 or GIFQ-160 videoendoscopes following existing protocol [29]. A total of 8 biopsies of approximately 2×2×2 mm in dimensions were obtained from the stomach (4 from the antrum, and 2 each from the corpus and fundus) and 2 from the distal esophagus 2 cm above the gastroesophogeal junction. Biopsy specimens were processed according to pre-established standardized protocols and reviewed by an experienced gastrointestinal histopathologist (Z.P.) designated to the study [20]. The pathologist reviewed all biopsy specimens and was blinded to questionnaire data and findings from the oral examination [20]. Parameters evaluated for gastric biopsies in cluded (a) degree of acute inflammation (b) atrophy, and (c) intestinal metaplasia. The degree of gastritis was scored using the updated Sydney System [30]. Participants were categorized according to the most advanced lesion found. Results from biopsies were classified as cases and non-cases: 1) cases had chronic atrophic gastritis, intestinal metaplasia, or dysplasia; and 2) non-cases had either chronic superficial gastritis or no evidence of gastric lesions.

The Institutional Review Boards of New York University School of Medicine and Bellevue Hospital Center approved all procedures involving human subjects. Written informed consent was obtained from all subjects at time of enrollment.

Overall, 283 eligible participants were approached, and 162 were recruited between April 2009 and April 2011. One hundred thirty one (131) subjects underwent an oral health clinical assessment and provided saliva and plaque samples for microbiological evaluation. Participants and non-participants were relatively similar in demographic distribution; roughly two thirds of non-participants were of Asian or Hispanic race/ethnicity, 60% were female, and their average age was 58 years. The top three reasons for declining participation (80%) included: not having time for the oral exam, not interested in the present research, and not wanting to participate in research studies in general. As of April 2012, complete data on selected oral pathogens was available from 119 participants (37 cases and 82 non-cases).

### Oral examination and collection of saliva and plaque sample

Details of the oral examination have been previously presented elsewhere [20]. Within 1–4 weeks following upper endoscopy, a dental hygienist calibrated in all assessments performed a comprehensive oral examination on all participants, and was blinded to biopsy results of gastroenterology. Periodontal assessments were standardized in a calibration session performed prior to the study and followed National Institute of Dental and Craniofacial Research diagnostic criteria, used previously in the Oral Health Survey of U.S. Adults [31]. Periodontal examinations involved assessments at six tooth sites (mesiobuccal, buccal, distobuccal, distolingual, lingual and mesiolingual) of all teeth present, using a manual North Carolina 15 periodontal probe [32]. Levels of clinical attachment loss (CAL), defined as the distance from the cemento-enamel junction to the free gingival margin in millimeters, and periodontal probing depth (PD), defined as the distance from the free gingival margin to the bottom of the pocket to the nearest whole millimeter, were recorded for each of the six tooth surfaces assessed. Bleeding on probing (BOP) was recorded dichotomously for each tooth surface and deemed positive if it occurred within 15 seconds after the assessment of probing depth.

All subjects were asked to chew a piece of paraffin wax and then to gently expectorate 2–5 ml of saliva directly into a sample collection tube, on ice. A total of 6 plaque samples were collected from each subject with sterile curettes; 4 supragingival plaque samples from the 1^st^ molar or most posterior tooth in each quadrant and 2 subgingival plaque samples from teeth exhibiting the deepest periodontal pockets. The plaque samples were immediately transferred to a pre-labeled sterile sample vial containing 200 µl TE buffer. Both saliva and plaques samples were vortex-mixed thoroughly for 30 sec, immediately placed into a container containing ice, and transferred to the laboratory within 1 h for further processing.

### Quantitative real-time PCR assays

Quantitative real-time PCR was performed to detect the presence/absence and to quantify targeted bacterial DNA in the saliva and plaque samples. Bacterial genomic DNA was isolated using the MasterPure DNA purification kit (Epicentre, Madison, WI) following previously established procedures [33]. After DNA isolation, the total genomic DNA concentration of each sample was quantified using a UV spectrophotometer at 260 nm and 280 nm (NanoDrop 2000C, Thermo Scientific, Wilmington, DE). The final concentration of each DNA sample was adjusted to 10 ng/µl for real-time qPCR. The qPCR reaction mixture contained a total volume of 25 µl, which consisted of 10× buffer, 2.5 mM/ea dNTPs, 10 pmole/µl primers, 5 U/µl Taq, 50 mm MgCl_2_, and 20 ng/µl DNA template. The amplifying reaction of DNA in a volume of 25 µl was composed of 1× QuantiTect SYBR Green PCR Master Mix (Qiagen), 10∼100 ng of total genomic DNA and 0.4 µM of each primer. The species-specific PCR primers used in this study are listed in **Table S1**. All PCR programs used for the amplifying reactions are listed in **Table S2**. Serial dilutions (10-fold) of standard DNA concentrations of *P. gingivalis* (ATCC33277), *T. forsythensis* (ATCC43037), *T. denticola* (ATCC35404), *A. actinomycetemcomitans* (ATCC29522), *S. mutans* (UA159), and *S. sobrinus* (OMZ176), were used in each reaction as external standards for absolute quantification of the targeted bacteria. DNA levels for all 6 bacterial species were measured in saliva samples, and 4 bacterial species relevant to periodontal disease (*A. actinomycetemcomitans, P. gingivalis, T. denticola*, *T*. forsythia) were measured in each plaque sample. We tested for the colonization of *S. sobrinus* and *S. mutans* in saliva rather than in plaque because the plaque samples were collected from specific tooth sites exhibiting the deepest periodontal pockets and may not truly reflect the overall prevalence of these cariogenic microorganisms in the oral cavity. Use of our real-time PCR method involving stimulated whole saliva has been a standard and well-documented procedure for evaluating prevalence of *S. sobrinus* and *S.* mutans [34], [35]. The analytical specificity of the fluorescence signal was determined on the basis of subsequent melting curve analysis. All reactions were carried out in duplicate, and the final analyses were based on the mean of the two reactions. Output data were analyzed using Opticon Monitor 2 software (MJ Research).

### Statistical Analyses

We first conducted descriptive analyses comparing participants with gastric precancerous lesions and those without gastric precancerous lesions in terms of socio-demographic, lifestyle characteristics, and periodontal disease indices, including CAL, BOP, and PD. We estimated Odds Ratios (ORs) for gastric precancerous lesions in relation to bacterial DNA levels in saliva samples and plaque samples by entering each measure as a continuous variable in serially adjusted models. DNA levels for each bacterial species measured in plaque were averaged for each individual (6 samples per person). Log-transformations of bacterial DNA levels were considered to postulate that disease risk is proportional to a power of dose in bacterial DNA levels (a log-linear relation between log odds and exposure), a relationship known to occur frequently from both human and animal studies [36]. To facilitate interpretation, we estimated ORs for gastric precancerous lesions in relation to a one standard deviation (SD) increase in log-transformed bacterial DNA levels of each of the bacterial species.

We also created a cumulative summary score of bacterial burden for bacterial species etiologically related to periodontal disease (*A. actinomycetemcomitans*, *P. gingivalis, T. denticola, T. forsythia* [21], [37]) and dental caries (*S. mutans and S. sobrinus* [25], [38]). The average colonization level of each species was natural log-transformed and standardized by dividing each person-specific value by the log-transformed population standard deviation (SD). One SD on the natural log scale was hence equivalent across microbes. The standardized values were then summed and grouped accordingly. We estimated ORs for gastric precancerous lesions in relation to the cumulative summary score entered as a continuous variable. ORs were adjusted for major risk factors of gastric cancer that may be related to oral health, and these factors included age, sex, ethnic background, smoking status, body mass index, *H. pylori* status, and educational attainment.

Since clinical periodontal disease is a manifestation of long-term infection of periodontal pathogens, we conducted stratified analyses to assess whether the association between periodontal disease-related pathogens and gastric precancerous lesions differs by periodontal indices, including CAL, BOP, and PD. The median values for percentage of tooth sites with BOP, CAL ≥3 mm, and PD≥3 mm were considered cut-points in stratified analyses. Significance of the interaction was tested using a cross-product term between a given periodontal disease index and a given periodontal disease bacterial species (or the cumulative burden of all four species), all entered as continuous variables in the model. Sensitivity analyses were performed using different cut-points for periodontal indices (e.g., percentage of sites with CAL≥5 mm and PD≥4 mm). Exploratory analyses were conducted to assess whether the exposure-disease association differed by *H. pylori* status.

We performed all statistical analyses using SAS 9.2 (SAS Institute, Inc., Cary, North Carolina). All tests were two sided and P<0.05 was considered significant.

## Results

Participants were largely of Hispanic ethnicity (45.4%), female (63.0%) and had a mean age of 57 years. Cases had a significantly higher prevalence of smoking (p = 0.02) and were more likely to have tested positive for *H. pylori* colonization (particularly with *cagA* strain, p = 0.07) than non-cases (**Table 1**). When we compared mean levels of periodontal indices by case group, we found a higher mean percentage of gingival bleeding sites (p = 0.04) among cases than non-cases. There were no other significant differences in periodontal disease indices by case status. As expected, we observed that the means of log-transformed periodontal pathogen DNA levels in plaque increased across tertiles of periodontal indices (**Table S3**). The DNA levels of periodontal pathogens were generally higher among those who did not floss as compared to those who did floss, although most did not reach statistical significance (data not shown).

**Table 1:** Distributions of participant characteristics by status of gastric precancerous lesions, n = 119.

Patient Characteristics	Gastric precancerous lesions		p-value^1^
	Cases (N = 37)	Non-cases (N = 82)	
**Sex, %**			
Men	32.4	39.0	0.49
Women	67.6	61.0	
**Age, years**			
Mean (SD)	58.2 (9.0)	56.9 (9.0)	0.44^2^
**Education, %**			
Less than high school	40.5	34.2	0.71
High school	27.0	34.2	
Some college or graduate	32.4	31.7	
**Race, %**			
Asian/Pacific Islander	35.1	26.8	0.41
Hispanic/African American	56.8	57.3	
White/Others	8.1	15.9	
**BMI, kg/m** 2			
Mean (SD)	26.8 (7.3)	26.2 (6.2)	0.69^2^
**Smoking Status, %**			
Ever	46.0	24.4	0.02
Never	54.1	75.6	
**Alcohol Consumption, %**			
Ever	41.7	42.7	0.91
Never	58.3	57.3	
**Tea Consumption, %**			
Yes	73.0	74.7	0.87
**Coffee Consumption, %**			
Yes	73.0	69.1	0.67
**Place of Birth, %**			
Foreign born	94.6	87.8	0.34^3^
***H.pylori*** ** status (in serum), %**			
Negative	31.4	53.3	0.07
Positive			
cagA negative	14.3	13.3	
cagA positive	54.3	33.3	
**% Bleeding sites**			
Mean (SD)	30.0 (21.3)	21.4 (17.0)	0.04^2^
**% sites with PD≥3 mm**			
Mean (SD)	21.9 (18.9)	20.8 (17.5)	0.76^2^
Mean PD (SD), mm	2.0 (0.6)	1.9 (0.5)	0.65^2^
**% sites with CAL≥3 mm**			
Mean (SD)	32.2 (22.0)	32.8 (21.2)	0.89^2^
Mean CAL (SD), mm	2.4 (0.9)	2.4 (0.7)	0.90^2^

PD = Pocket Depth, CAL = Clinical Attachment Loss.

1 p-value by chi-square test.

2 p-value for t-test.

3 p-value by two-sided fisher exact test.

Age and gender adjusted mean bacterial DNA levels in either saliva or plaque samples were not significantly different by case status (**Table 2**). Results from multivariate logistic regression models using log-transformed bacterial DNA levels appear generally similar. In saliva, there was no evidence that levels of caries-related bacteria were related to gastric precancerous lesions. For periodontal disease-related bacteria, DNA levels for *T. forsythia* were significantly inversely associated with gastric precancerous lesions in a model adjusted by age and gender (OR = 0.59, 0.40–0.88) and in the fully adjusted model (OR = 0.61, 0.38–0.98). There was no evidence that levels of other periodontal disease-related bacteria in saliva were related to gastric precancerous lesions. In plaque, however, there was no evidence of an association between DNA levels for *T. forsythia*. *A. actinomycetemcomitans* and *T. denticola* in plaque samples were positively associated with gastric precancerous lesions (ORs for one SD increase in log-transformed bacterial levels: 1.36, p = 0.17 and 1.34, p = 0.23, respectively; **model 2**). However, these associations did not reach statistical significance. Using the summary measure of bacterial burden in plaque, we similarly found a positive albeit non-significant association between one SD increase in log-transformed bacterial burden level and presence of gastric precancerous lesions in a fully adjusted multivariable model (OR = 1.22, 95% confidence interval: 0.77–1.92). Sensitivity analyses were performed using different cut-points for periodontal indices (e.g., percentage of sites with CAL≥5 mm and PD≥4 mm). However, the results were similar and are not shown here.

**Table 2:** Adjusted means and odds ratios for gastric precancerous lesions in relation to bacterial DNA levels, n = 119.

Bacterial DNA levels	Adjusted means^1^ (SE)			ORs^2^ for gastric precancerous lesions (95% CI)	
	Cases N = 37	Non-cases N = 82	P-value	Model 1	Model 2
Saliva					
*A. actinomycetemcomitans (Aa)*	2.0 (0.9)	0.9 (0.6)	0.30	1.14 (0.78–1.67)	1.12 (0.73–1.74)
*P. gingivalis* 3 *(Pg)*	2.1 (1.1)	2.6 (0.7)	0.66	0.98 (0.66–1.45)	1.06 (0.61–1.83)
*T. denticola (Td)*	4.3 (8.9)	15.4 (5.9)	0.29	0.95 (0.64–1.41)	1.10 (0.67–1.79)
*T. forsythia* 4 *(Tf)*	2.1 (7.0)	6.7 (4.7)	0.58	0.59 (0.40–0.88)	0.61 (0.38–0.98)
*S. sobrinus (Sb)*	3.1 (2.0)	0.5 (1.3)	0.28	1.09 (0.73–1.61)	0.98 (0.78–1.24)
*S. mutans (Sm)*	4.3 (2.8)	8.0 (1.9)	0.27	0.89 (0.60–1.33)	0.98 (0.84–1.15)
Cumulative burden^5^ (*Sb, Sm*)	−1.8 (0.3)	−1.8 (0.2)	0.92	0.98 (0.65–1.47)	0.94 (0.60–1.49)
Cumulative burden^5^ (*Aa, Pg, Td, Tf*)	−1.2 (0.5)	−0.7 (0.3)	0.43	0.85 (0.57–1.27)	0.91 (0.56–1.49)
Plaque					
*A. actinomycetemcomitans (Aa)*	1.6 (1.5)	1.5 (1.0)	0.96	1.28 (0.87–1.89)	1.36 (0.87–2.12)
*P. gingivalis* 3 *(Pg)*	6.2 (3.8)	9.6 (2.5)	0.45	0.99 (0.67–1.47)	1.12 (0.67–1.88)
*T. denticola* 4 *(Td)*	1.3 (0.9)	1.5 (0.6)	0.90	1.15 (0.77–1.71)	1.34 (0.83–2.15)
*T. forsythia* 6 *(Tf)*	3.9 (1.7)	5.0 (1.1)	0.59	0.76 (0.52–1.11)	0.90 (0.58–1.38)
Cumulative burden^5^ (Aa, Pg, Td, Tf)	0.6 (0.5)	0.5 (0.3)	0.88	1.03 (0.69–1.54)	1.22 (0.77–1.92)

1 Means of absolute bacterial counts (ng/µl) adjusted for age and sex.

2 ORs estimated in relation to a standard deviation increase in the log-transformed bacterial DNA levels. Model 1 was adjusted for age and sex. Model 2 was further adjusted for race, smoking status, educational attainment, BMI, and *H. pylori* status.

3 Bacterial counts in 1×10**^2^** ng/µl.

4 Bacterial counts in 1×10**^4^** ng/µl.

5 Cumulative burden is the sum of the log-transformed and standardized values of bacterial counts.

6 Bacterial counts in 1×10**^3^** ng/µl.

To assess the effect of more specific long-term infection, we further investigated whether the association between periodontal pathogens in plaque and gastric precancerous lesions differed by periodontal disease indices (**Table 3**). The odds ratio for gastric precancerous lesions associated with one SD increase in levels of *A. actinomycetemcomitans* was greater (OR: 2.51, 95% CI = 1.13–5.56) among participants with a higher percentage of tooth sites (≥ the median) with PD≥3 mm, after controlling for gender, age, race, smoking status, educational attainment, BMI, and *H. pylori* status. By contrast, this effect was considerably attenuated among those with a lower percentage of sites (< the median) with PD≥3 mm (OR = 0.86, 95% CI = 0.43–1.72). The association between *A. actinomycetemcomitans* and gastric precancerous lesions was also elevated among individuals with a higher percentage of sites with CAL≥3 mm (OR = 1.92, 95% CI = 0.96–3.85) and a higher percentage of BOP (OR = 1.38, 95% CI = 0.72–2.65). Similar patterns of associations were evident for other periodontal disease-related pathogens. When the cumulative summary score of all four bacterial species was used in the analyses, we observed that the association between one SD increase in the summary score and gastric precancerous lesions among participants with higher levels of periodontal disease (i.e., percentage of sites with BOP, PD≥3 mm, or CAL≥3 mm) was significantly or marginally significantly stronger than those with lower levels of periodontal disease (p-values for interaction ranged from 0.03–0.06). Lastly, in exploratory analyses, we investigated whether the associations between oral pathogens and gastric precancerous lesions differed by *H. pylori*. The associations were not appreciably different by *H. pylori* status and the results are therefore not shown.

**Table 3:** Adjusted odds ratios for gastric precancerous lesions in relation to selected periodontal pathogen DNA levels and stratified by periodontal disease severity^1^.

		Odds ratios^2^ for gastric precancerous lesions (95% CI)				
Stratified Analyses	Cases/non-cases	*A. actinomycetemcomitans*	*P. gingivalis*	*T. denticola*	*T. forsythia*	Cumulative bacterial burden^3^
% Bleeding sites						
≥18.8	22/37	1.38 (0.72–2.65)	1.19 (0.60–2.37)	1.30 (0.67–2.52)	1.43 (0.72–2.84)	1.42 (0.75–2.70)
<18.8	15/45	0.42 (0.11–1.61)	0.63 (0.23–1.76)	1.12 (0.46–2.75)	0.27 (0.09–0.83)	0.39 (0.13–1.13)
P for interaction^4^		0.30	0.31	0.39	0.06	0.06
% PD≥3 mm						
≥16.7	17/40	2.51 (1.13–5.56)	1.66 (0.75–3.64)	1.61 (0.79–3.30)	1.36 (0.69–2.66)	2.00 (0.97–4.13)
<16.7	20/42	0.86 (0.43–1.72)	0.88 (0.43–1.87)	1.12 (0.55–2.31)	0.54 (0.26–1.13)	0.73 (0.35–1.51)
P for interaction^4^		0.08	0.18	0.16	0.07	0.04
% CAL ≥3 mm						
≥29.2	18/39	1.92 (0.96–3.85)	1.47 (0.61–3.56)	1.23 (0.58–2.62)	1.08 (0.57–2.07)	1.54 (0.75–3.19)
<29.2	19/43	0.97 (0.43–2.18)	1.04 (0.45–2.36)	1.72 (0.74–3.98)	0.68 (0.31–1.51)	1.03 (0.46–2.32)
P for interaction^4^		0.14	0.03	0.27	0.04	0.03

PD = Pocket Depth, CAL = Clinical Attachment Loss.

1 Cut-points for periodontal disease severity were determined at the median.

2 ORs estimated in relation to a standard deviation increase in the log-transformed bacterial DNA levels and adjusted for gender, age, race, smoking status, educational attainment, BMI, and *H. pylori* status.

3 Cumulative bacterial burden is a summary measure estimated by standardizing each bacterial DNA level (dividing each person-specific value by the log-transformed population standard deviation) and summing these values across all four species.

4 P-value for the cross-product term of each log-transformed bacterial DNA level and each periodontal index entered as continuous variables.

## Discussion

Previous studies have reported positive associations between poor oral health and tooth loss with gastric cancer [15]–[18]. Ours is the first to investigate associations between DNA levels of oral pathogens and presence of gastric precancerous lesions. In the present study, there was no consistent association between DNA levels of selected bacterial species etiologically related to periodontal disease and presence of gastric precancerous lesions. Among those with higher levels of periodontal disease, periodontal pathogens measured in plaque were positively related to presence of gastric precancerous lesions, independent of gender, age, race, smoking status, educational attainment, BMI, and *H. pylori* status. The findings support the role of specific manifestations of periodontal disease and periodontal infection in the development of gastric cancer.

It has been proposed that bacterial infections are linked with gastric cancer through the induction of systemic or gastric chronic inflammation [39]. In the classic example of *H. pylori*, infection with one pathogen alone triggers chronic inflammation and leads to gastric non-cardia adenocarcinoma [40]–[42]. Periodontal pathogen *A. actinomycetemcomitans* has been associated with increased secretion of interleukins 1α and 1β and tumor necrosis factor α, cytokines that are involved in the inflammatory response [43], [44]. High serum titre to *P. gingivalis* and the presence of periodontal disease are independently related to high C-reactive protein levels [45]. Recently, polymorphisms in the gene of Interleukin 6, a pro-inflammatory cytokine, were associated with *A. actinomycetemcomitans*, *P. gingivalis, T. denticola*, and *T. forsythia* [46]. Periodontal disease is a set of inflammatory conditions that leads to destruction of the supporting alveolar bone, connective tissue, and eventual tooth loss [47]. In this study, DNA levels for *T. forsythia* in saliva were significantly inversely associated with gastric precancerous lesions. However, there was no evidence for this in plaque. A non-significant positive association was observed between other periodontal disease-related bacteria measured in plaque and gastric precancerous lesions. Among individuals with more severe symptoms of periodontal disease, we observed consistent and positive associations between periodontal pathogens and gastric precancerous lesions. It is possible that higher levels of periodontal pathogens in individuals with a higher percentage of BOP and deeper periodontal pockets are indicative of more specific long-term infection that has a more pronounced effect on inflammation. There is also some evidence to show that higher levels of periodontal pathogens are associated with more aggressive forms of periodontitis [48], [49]. Importantly, the positive associations among those with periodontal disease were independent of *H. pylori* and other established risk factors. If results are confirmed in future studies, the combined status of periodontal disease indices and their corresponding pathogens may be considered risk factors for gastric precancerous lesions and gastric cancer. Since bacterial profiles and oral health conditions are modifiable, identification of bacterial risk factors of malignancy could have important implications for cancer prevention.

In the present study, the positive associations between selected pathogens or their cumulative burden score and gastric precancerous lesions were similar across bacterial species among participants with higher levels of periodontal disease indices. The findings suggest a potential role of multiple periodontal disease-related bacteria. An emerging concept in microbial pathogenesis is the notion that the community functions as a pathogen and contributes to disease development collectively, rather than through the action of one specific member alone [50], [51]. Future studies are needed to comprehensively investigate the role of other oral pathogens or oral microbiomes related to inflammation and periodontal disease in gastric cancer development.

There were several strengths of the present study. First, clinical indices of periodontal disease were measured objectively in a consistent fashion for all participants. Second, the availability of data on important risk factors of gastric cancer and potential confounders enhanced the ability to control for potential confounding and strengthened the validity of the study findings. Third, the bacterial specificity of our findings lends further support to the role of periodontal disease in the development of gastric cancer. In our study, no apparent relationship was evident between caries-related bacteria and gastric precancerous lesions. This is consistent with our previous study, which found no association between any clinical measure of caries (number of decayed, missing, or filled teeth/tooth surfaces) and gastric precancerous lesions^20^. In addition, the association between selected periodontal disease bacteria and gastric precancerous lesions were more evident for bacterial levels measured in pooled plaque compared with levels measured in whole saliva. Levels of microorganisms in subgingival plaque may be less diluted than in saliva and therefore more sensitive to detection by real-time qPCR.

Several potential limitations, however, should also be noted. First, the study had a small sample size and therefore we would have limited power to assess associations between oral pathogens and gastric precancerous lesions if oral pathogens were treated as categorical variables. We also investigated a number of comparisons in the study. Since p-values from the various models were not independent and the work was hypothesis-oriented, we adjusted p-values using the Benjamini–Hochberg false discovery rate (FDR) method to account for multiple comparisons [52]. After adjustment for FDR, the adjusted p-values for the interaction effects of % CAL≥3 mm with Pg and Tf remained marginally significant (p = 0.08), while the significance of the other interactions did not (FDR-adjusted p-value >0.15). Moreover, the inverse association of *T. forsythia* with gastric precancerous lesions was no longer significant (FDR-adjusted p-value = 0.25). Larger studies are needed to confirm our findings and to further investigate the full dose-response relationships. Second, the study is cross-sectional and precludes any definitive statement regarding a causal association. However, DNA levels of oral pathogens and periodontal disease indices were measured objectively and were blinded to case status. Although we cannot also exclude the possibility of potential selection bias, it is not likely that cases with severe periodontal disease preferentially volunteered to be included in the study, since the hypotheses were not known to participants and were unaware of their lesion status at the time of recruitment for the oral health examination.

In conclusion, we found an overall non-significant positive association between DNA levels of selected oral pathogens related to periodontal disease, and the associations were stronger among individuals with higher levels of periodontal disease indices. Our findings highlight the role of periodontal infection in the development of gastric cancer.

## Supporting Information

Table S1 **Species-specific primers that were utilized for real-time quantitative PCR (qPCR).**
(DOCX)

Table S2 **Programs that were utilized for real-time quantitative PCR (qPCR).**
(DOCX)

Table S3 **Means of log-transformed bacterial DNA values across tertiles of periodontal disease indices.**
(DOCX)
