# Supplementary material for: Association between Selected Oral Pathogens and Gastric Precancerous Lesions
Source: PLoS One. 2013 Jan 7;8(1):e51604. doi: 10.1371/journal.pone.0051604 (PMC3538744; doi:10.1371/journal.pone.0051604)
Supplement: Table S1 — Species-specific primers that were utilized for real-time quantitative PCR (qPCR). (DOCX) [file pone.0051604.s001.docx]

**Table S1**. Species-specific primers that were utilized for real-time quantitative PCR (qPCR)

| **Selected oral pathogen** | **Primer pairs (5’~3’)** | **Amplicon length**  **(bp)** | **References** |
| --- | --- | --- | --- |
| Periodontal disease pathogens |  |  |  |
| *P. gingivalis*  Gram-negative anaerobe | AGG CAG CTT GCC ATA CTG CG  ACT GTT AGC AAC TAC CGA TGT | 404 | Byrne, SJ et al. [1] |
| *T. forsythensis*  Gram-negative anaerobe | TAC AGG GGA ATA AAA TGA GAT ACG  TTC ACC GCG GAC TTA ACA GC | 250 | Tran, S.D. and Rudney, J.D. [2]  Rudney J.D., Chen R, and Pan, Y. [3] |
| *T. denticola*  Gram-negative anaerobe | TAA TAC ATG TGC TCA TTT ACA T  TCA AAG CAT TCC CTC TTC TTC TTA | 316 | Byrne, SJ, et al. [[1]](#_ENREF_1) |
| *A. actinomycetemcomitans*  Gram-negative anaerobe | ATT GGG GTT TAG CCC TGG T  GGC ACA AAC CCA TCT CTG A | 250 | Rudney J.D., Chen R, and Pan, Y.[3] |
|  |  |  |  |
| Dental caries pathogens |  |  |  |
| *S. mutans*  Gram-positive anaerobe | TCG CGA AAA AGA TAA ACA AAC A  GCC CCT TCA CAG TTG GTT AG | 479 | Chen, Z. et al. [4] |
| *S. sobrinus*  Gram-positive anaerobe | TTC AAA GCC AAG ACC AAG CTA GT  CCA GCC TGA GAT TCA GCT TGT | 88 | Yoshida, A., et al. [5] |

1. Byrne SJ, Dashper SG, Darby IB, Adams GG, Hoffmann B, et al. (2009) Progression of chronic periodontitis can be predicted by the levels of Porphyromonas gingivalis and Treponema denticola in subgingival plaque. Oral Microbiol Immunol 24: 469-477.

2. Tran SD, Rudney JD. (1999) Improved multiplex PCR using conserved and species-specific 16S rRNA gene primers for simultaneous detection of Actinobacillus actinomycetemcomitans, Bacteroides forsythus, and Porphyromonas gingivalis. J Clin Microbiol 37: 3504-3508.

3. Rudney JD, Chen R, Pan Y (2003) Endpoint quantitative PCR assays for Bacteroides forsythus, Porphyromonas gingivalis, and Actinobacillus actinomycetemcomitans. J Periodontal Res 38: 465-470.

4. Chen Z, Saxena D, Caufield PW, Ge Y, Wang M, et al. (2007) Development of species-specific primers for detection of Streptococcus mutans in mixed bacterial samples. FEMS Microbiol Lett 272: 154-162.

5. Yoshida A, Suzuki N, Nakano Y, Kawada M, Oho T, et al. (2003) Development of a 5' nuclease-based real-time PCR assay for quantitative detection of cariogenic dental pathogens Streptococcus mutans and Streptococcus sobrinus. J Clin Microbiol 41: 4438-4441.
