# Supplementary material for: Association between Selected Oral Pathogens and Gastric Precancerous Lesions
Source: PLoS One. 2013 Jan 7;8(1):e51604. doi: 10.1371/journal.pone.0051604 (PMC3538744; doi:10.1371/journal.pone.0051604)
Supplement: Table S2 — Programs that were utilized for real-time quantitative PCR (qPCR). (DOCX) [file pone.0051604.s002.docx]

**Table S2**. Programs that were utilized for real-time quantitative PCR (qPCR)

| **Bacteria** | **Program** | | | | |
| --- | --- | --- | --- | --- | --- |
|  | **Preincubation** | **Cycles** | **Denaturation** | **Annealing** | **Extension** |
| *P. gingivalis* |  | 40 |  | 60 ℃ 30 s |  |
| *T. forsythensis* |  | 45 |  | 55 ℃ 30 s |  |
| *T. denticola* | 95 ℃15 min | 45 | 94 ℃ 15 s | 55 ℃ 30 s | 72 ℃ 30 s |
| *A. actinomycetemcomitans* |  | 40 |  | 58 ℃ 30 s |  |
| *S. mutans* |  | 45 |  | 56 ℃ 30 s |  |
| *S. sobrinus* |  | 45 |  | 58 ℃ 30 s |  |
