# Supplementary material for: Association between Selected Oral Pathogens and Gastric Precancerous Lesions
Source: PLoS One. 2013 Jan 7;8(1):e51604. doi: 10.1371/journal.pone.0051604 (PMC3538744; doi:10.1371/journal.pone.0051604)
Supplement: Table S3 — Means of log-transformed bacterial DNA values across tertiles of periodontal disease indices. (DOCX) [file pone.0051604.s003.docx]

**Table S3.** Means of log-transformed bacterial DNA values across tertiles of periodontal disease indices

|  | **Tertile 1** | **Tertile 2** | **Tertile 3** |
| --- | --- | --- | --- |
| *A. actinomycetemcomitans* |  |  |  |
| % bleeding sites | -3.77 | -2.98 | -2.72 |
| % PD ≥ 3 mm | -3.36 | -3.36 | -2.73 |
| % CAL ≥ 3mm | -3.40 | -3.05 | -3.00 |
| *P. gingivalis* |  |  |  |
| % bleeding sites | -1.13 | 1.86 | 4.68 |
| % PD ≥ 3 mm | -0.26 | 0.30 | 5.39 |
| % CAL ≥ 3mm | -0.19 | 1.58 | 4.01 |
| *T. denticola* |  |  |  |
| % bleeding sites | -1.01 | 0.97 | 2.59 |
| % PD ≥ 3 mm | -0.56 | 0.13 | 2.99 |
| % CAL ≥ 3mm | -0.34 | 0.97 | 1.91 |
| *T. forsythia* |  |  |  |
| % bleeding sites | 4.51 | 5.33 | 7.01 |
| % PD ≥ 3 mm | 4.00 | 5.47 | 7.39 |
| % CAL ≥ 3mm | 4.28 | 6.01 | 6.55 |
